# The emerging role of neutrophil extracellular traps in severe acute respiratory syndrome coronavirus 2 (COVID-19)

**DOI:** 10.1038/s41598-020-76781-0

**Published:** 2020-11-12

**Authors:** Angélica Arcanjo, Jorgete Logullo, Camilla Cristie Barreto Menezes, Thais Chrispim de Souza Carvalho Giangiarulo, Mirella Carneiro dos Reis, Gabriellen Menezes Migliani de Castro, Yasmin da Silva Fontes, Adriane Regina Todeschini, Leonardo Freire-de-Lima, Debora Decoté-Ricardo, Antônio Ferreira-Pereira, Celio Geraldo Freire-de-Lima, Shana Priscila Coutinho Barroso, Christina Takiya, Fátima Conceição-Silva, Wilson Savino, Alexandre Morrot

**Affiliations:** 1 Medical Biochemistry Institute, Federal University of Rio de Janeiro, Rio de Janeiro, Brazil; 2 Carlos Chagas Filho Biophysics Institute, Federal University of Rio de Janeiro, Rio de Janeiro, Brazil; 3 Tuberculosis Research Laboratory, Faculty of Medicine, Federal University of Rio de Janeiro, Rio de Janeiro, Brazil; 4 Molecular Biology Laboratory, Institute of Biomedical Research, Marcílio Dias Naval Hospital, Navy of Brazil, Rio de Janeiro, Brazil; 5 Parasitic Diseases Division, Marcílio Dias Naval Hospital, Navy of Brazil, Rio de Janeiro, Brazil; 6 Microbiology Institute, Federal University of Rio de Janeiro, Rio de Janeiro, Brazil; 7 Veterinary Institute, Federal Rural University of Rio de Janeiro, Rio de Janeiro, Brazil; 8 Immunoparasitology Laboratory, Oswaldo Cruz Foundation, Oswaldo Cruz Institute/FIOCRUZ, Bld. Leônidas and Maria Deane/Room 406C, Av. Brazil 4365, Manguinhos, Rio de Janeiro, RJ Brazil; 9 Laboratory on Thymus Research, Oswaldo Cruz Institute, Oswaldo Cruz Foundation, Rio de Janeiro, Rio de Janeiro Brazil; 10 National Institute of Science and Technology on Neuroimmunomodulation—INCT-NIM, Oswaldo Cruz Institute, Oswaldo Cruz Foundation, Rio de Janeiro, Rio de Janeiro Brazil; 11 Rio de Janeiro Research Network on Neuroinflammation, Oswaldo Cruz Institute, Oswaldo Cruz Foundation, Rio de Janeiro, Rio de Janeiro Brazil

**Keywords:** Infection, SARS-CoV-2

## Abstract

The novel coronavirus SARS-CoV-2 causes COVID-19, a highly pathogenic viral infection threatening millions. The majority of the individuals infected are asymptomatic or mildly symptomatic showing typical clinical signs of common cold. However, approximately 20% of the patients can progress to acute respiratory distress syndrome (ARDS), evolving to death in about 5% of cases. Recently, angiotensin-converting enzyme 2 (ACE2) has been shown to be a functional receptor for virus entry into host target cells. The upregulation of ACE2 in patients with comorbidities may represent a propensity for increased viral load and spreading of infection to extrapulmonary tissues. This systemic infection is associated with higher neutrophil to lymphocyte ratio in infected tissues and high levels of pro-inflammatory cytokines leading to an extensive microthrombus formation with multiorgan failure. Herein we investigated whether SARS-CoV-2 can stimulate extracellular neutrophils traps (NETs) in a process called NETosis. We demonstrated for the first time that SARS-CoV-2 in fact is able to activate NETosis in human neutrophils. Our findings indicated that this process is associated with increased levels of intracellular Reactive Oxygen Species (ROS) in neutrophils. The ROS-NET pathway plays a role in thrombosis formation and our study suggest the importance of this target for therapy approaches against disease.

## Introduction

There is global health emergency on the threat of a rapidly moving pathogenic SARS-coronavirus 2 (SARS-CoV-2) causing COVID-19, highly lethal pandemic of a respiratory pathogen with the potential for killing millions of people^1^. Coronaviruses are single-stranded positive-sense RNA viruses belonging to the Coronaviridae family. These viruses mainly infect animals, including mammals. Some recent human coronavirus infections have resulted in lethal epidemics, which include the endemic Severe Acute Respiratory Syndrome (SARS) and Respiratory Syndrome in the Middle East (MERS). In humans, coronavirus infection usually cause mild respiratory infections, like those seen in the common cold including fever, cough, and shortness of breath^2^. The majority of those infected in the COVID-19 pandemic are asymptomatic, however about 20% of the patients can progress clinically to acute respiratory distress syndrome (ARDS) in severe patients with an increased risk of vascular hyperpermeability, pneumonia, sepsis, ultimately leading to death^1,2^.

The overproduction of early response proinflammatory cytokines (TNFα, IL-6, IL-1β and IFN-γ) during innate response against SARS-CoV-2 virus results in a cytokine storm, leading to a diffuse alveolar damage evolving with shock and pulmonary dysfunction^3^. The disease has been revealed as a systemic disease, the increased expression of ACE2 in patients with comorbidities may represent a propensity for an increased viral load of infection and the spread of the virus to extrapulmonary tissues^4–6^. This systemic virus dissemination brings complications to different organs in patients who evolve for the severe clinical form, which may affect central nervous system, eyes, heart and gut, leading to multi-organ dysfunction and extensive microthrombus formation with multiorgan failure^7^. Evidence has shown that patients recovering from SARS-CoV-2 infection show signs of antiviral T and B cell adaptive immunity, with clonal expansion and activation^8^.

However, the deregulated innate immune response and consequent viral dissemination in patients susceptible to SARS-CoV-2 infection also contributes to events driving a nonresponsive state of T cell responses. Lung-infiltrating CD8^+^ T cells from severe COVID-19 patients potently exhibit hallmarks of T cell exhaustion, up-regulating PD-1 and Tim-3 markers^9^. Analysis of highly expanded T cell populations obtained from peripheral blood of severe COVID-19 patients indicate a pronounced expression of MKI67 and TYMS markers of terminally exhausted T cells^10^. The failure of an efficient immunological response early in disease may lead to persistent viral antigen thus contributing to the clinical presentation of patients with severe SARS-CoV-2 infections. Histopathologic analysis of COVID-19 patients have revealed the viral persistence in severe clinical forms^11,12^. In the absence of a protective immune response, the burst of load promotes sustained levels of neutrophils described as a clinical hallmark of COVID-19. In fact, severe patients show higher neutrophil to lymphocyte ratio (NLR), which is suggested as a predictive marker of death^13^.

A mounting body of evidence shows that severe clinical forms are associated to coagulation dysfunction markers, mainly D-dimer, platelet reduction, increase in prothrombin time as well as fibrin degradation products^7^. These factors suggest hyperactivity of the coagulation system in organ failure and death. All these findings indicate a potential cross-talk with neutrophil extracellular traps (NETs). NETs are structures made up of intracellular components released by activated neutrophils that discharge DNA, histones and proteins derived from intracellular granules. This process is called NETosis and plays a role in controlling pathogens, but also has a dentrimental effect in cardiovascular and pulmonary diseases^14^. Intravascular NETosis in COVID-19 infection could play a role in the vasculature complications, where thrombotic disease can drive organ damage. Therefore, in this report we investigated whether SARS-CoV-2 leads to NETs formation and the production of reactive oxygen species by neutrophils that could contribute to disease pathogenesis.

## Material and methods

### Human samples and SARS-CoV-2 isolation

Serum samples from 20 hospitalized COVID-19 patients in the acute phase of infection were used in this study (Table 1). Blood were collected into a serum vacutainer tube and allow it to clot at room temperature for 1 h. The criteria for confirmed cases with acute SARS-CoV-2 infection included positive result of the nucleic acid sequence of SARS-CoV-2 by real-time RT-PCR from nasopharyngeal swab samples based on FDA-approved RNA testing. Severe COVID-19 patients were clinically classified as having fever, respiratory infection, respiratory rate of 23 incursions/minute, dyspnea and oxygen saturation < 93% at room air. Healthy volunteers and severe COVID-19 patients were recruited from Hospital Naval Marcílio Dias, Rio de Janeiro, Brazil. COVID-19 infected patients were selected with ages ranging from 18 to 83 years (Table 1). Donors, age and sex matched-non-infected controls were included in the study. SARS-CoV-2 virus (provided by Amilcar Tanury, Laboratory of Molecular Virology, Federal University of Rio de Janeiro) was isolated from nasal swab specimens obtained from severe COVID-19 patient. Infection with SARS-CoV-2 was confirmed by performing real-time reverse transcription polymerase chain reaction (RT-PCR) assay, followed by viral N gene sequencing, and virus isolation on Vero E6 cell line. The research was approved by the Research Ethics Committee (CEP) from Brazilian National Health Council and all patients signed a free and informed consent form in accordance with current legislation and the relevant ethical regulations approved by the Hospital Naval Marcílio Dias (CAAE # 31642720.5.0000.5256) and Hospital Universitário Clementino Fraga Filho (CAAE # 30424020.0.0000.0008).

**Table 1:** Clinical data of COVID-19 severe acute patients whose sérum samples were included in the study.

Patient	Gender	Age	Symptoms	RT-qPCR (Swab)	Comorbidities	Respiratory status	Clinical classification	Hospitalization (days)	Outcome
P#1	Female	72	Fever, cough, shortness of breath, diarrhea	Positive	Hypertension, diabetes, occlusive peripheral arterial disease	Mechanical ventilation	Severe	12	Death
P#2	Male	76	Fever, acute viral nasopharyngitis, distress	Positive	Hypertension, Chronic arrhythmia	Mechanical ventilation	Severe	10	Death
P#3	Male	73	Fever, smell and/or taste loss, nausea, cough, fatigue, shortness of breath, diarrhea	Positive	Hypertension, diabetes, obesity, chronic obstructive pulmonary disease	Mechanical ventilation	Severe	26	Death
P#4	Female	74	Fever, cough, shortness of breath, distress	Positive	Cancer, chronic arrhythmia	Mechanical ventilation	Severe	15	Death
P#5	Female	71	Fever, cough, shortness of breath	Positive	Hypertension, diabetes, obesity, dyslipidemia	Mechanical ventilation	Severe	21	Death
P#6	Male	83	Cough, shortness of breath, diarrhea	Positive	Diabetes	Mechanical ventilation	Severe	12	Death
P#7	Female	78	Fever, cough, shortness of breath, diarrhea	Positive	Hypertension, diabetes, coronary disease	Mechanical ventilation	Severe	20	Death
P#8	Male	76	Fever	Positive	kidney disease	Mechanical ventilation	Severe	NI	Death
P#9	Male	63	Fever, nausea, cough, shortness of breath, diarrhea, muscle/joint pain	Positive	Hypertension	Mechanical ventilation	Severe	89	Hospital discharge
P#10	Male	67	Shortness of breath, altered state of consciousness	Positive	Hypertension	Oxygen therapy	Severe	8	Hospital discharge
P#11	Male	63	Fever, cough, fatigue, shortness of breath, diarrhea	Positive	Hypertension, diabetes, obesity	Oxygen therapy	Severe	10	Hospital discharge
P#12	Male	76	NI	Positive	Diabetes, kidney disease	Mechanical ventilation	Severe	NI	Death
P#13	Female	55	Fever, cough, acute viral nasopharyngitis, shortness of breath	Positive	Hypertension, obesity	Mechanical ventilation	Severe	50	Hospital discharge
P#14	Female	78	Nausea	Positive	Hypertension, diabetes, obesity	Mechanical ventilation	Severe	14	Death
P#15	Male	67	Fever, smell and/or taste loss	Positive	Diabetes, cancer	Mechanical ventilation	Severe	NI	Hospital discharge
P#16	Female	18	Fever, shortness of breath	Positive	Obesity	Mechanical ventilation	Severe	11	Hospital discharge
P#17	Female	75	Fever, cough, fatigue, shortness of breath, diarrhea	Positive	Hypertension, diabetes	Mechanical ventilation	Severe	21	Death
P#18	Male	60	Fever, shortness of breath, diarrhea	Positive	Hypertension, kidney disease, obesity	Mechanical ventilation	Severe	NI	NI
P#19	Male	43	Fever, nausea, cough, fatigue, shortness of breath, diarrhea	Positive	N/A	Mechanical ventilation	Severe	13	Death
P#20	Male	67	distress	Positive	Congestive heart failure	Mechanical ventilation	Severe	33	Death

### NETosis and phagocytosis assay

Neutrophils were isolated from peripheral blood from the collection of 20 mL of heparinized blood. The blood collected was slowly placed in a 50 mL tube containing 1:2 Ficoll, the gradient was centrifuged for 30 min at room temperature without braking and without acceleration (400*g*). After centrifugation, the upper part containing mononuclear cells was discarded and the neutrophils were collected with a Pasteur pipette and, after lysis of the red blood cells, the leukocytes were resuspended in RPMI medium, counted and adjusted for each experimental condition. For NETosis assays, neutrophils were suspended in RPMI medium containing 1% nutridoma and adjusted to 1 × 10^5^ neutrophils/mL. We stimulated or not with virus MOI 9.0, PMA 100 nM, serum from normal and severely infected patients, LPS 10 ng/mL. PMA and LPS (*E. coli* O55:B5) were purchased from Sigma-Aldrich and Invivogen (San Diego, CA), respectively. Samples were then incubated for 90 min at 37 °C/5% CO_2_^15^, the cells were centrifuged at 4 °C/1600 RPM for 6 min, and the supernatants were collected for extracellular DNA measurement. Then, 25 µL of the supernatants were added to 50 µL of tris–EDTA buffer (1 mM at pH 8.0) and 25 µL of Quant-it Picogreen dsDNA reagent (Thermofisher), a highly sensitive fluorescent DNA dye. The reading was made using a 528 nm emission filter, with 485 nm excitation in a microplate reader (Spectramax M3). For phagocytosis assays, 5 × 10^4^ neutrophils were cultured in black 96-well plate treated with 0.001% L-polylysine and incubated in at 37 °C/5% CO_2_ for 90 min with or without stimuli (COVID-19 virus at MOI 9.0; sera from either normal or severe infected patients at 10%; PMA 100 nM). In specific experiments, viral loads of 3.0 MOI and 1.0 MOI were applied. Phagocytic activity was analyzed from the incubation of Dextran beads conjugated to tetramethyl rhodamine (2,000,000 MW, Sigma-Aldrich) added to the cells (200 µg/mL) in the presence of the givrn stimuli. After 90 min, the wells were washed, and the fluorescence incorporated into the cells was read in a fluorometer with 555 nm excitation and 580 nm emission.

### Dosage of reactive oxygen species

For the detection of intracellular Reactive Oxidizing Species (ROS), the dichlorodihydrofluorescein diacetate probe (H2DCFDA, Invitrogen) was used. This probe reacts with free radicals in general and emits green fluorescence. For the assays, the 5 × 10^4^ neutrophils were cultured in a 96-well plate treated with 0.001% L-polylysine (Sigma-Aldrich) and incubated at 37 °C/5% CO_2_ for 90 min in the presence or absence of stimuli: COVID-19 virus (MOI 9.0); 10% serum from normal or severely infected patients; PMA 100 nM. Quantification of ROS was done by adding 20 μM H2DCFDA probe (Invitrogen) 15 min before the end of incubation. The plates were read at 530 nm emission and 485 nm excitation using a fluorimeter (Spectramax M3).

### Quantification of IL-8

Interleukin 8 (IL-8) was quantified from neutrophil culture supernatants (5 × 10^4^/50 µL) in the presence or absence of SARS-CoV-2 virus (MOI 9.0) according to the manufacturer's recommendations (Quantikine Elisa/R&D System). In the assay 100 µL of assay diluent were added to the wells (in 96-well plates) along with the standards, samples and negative control. After 2 h of incubation at 37 °C/5% CO_2_, the wells were washed 4 times with 200 µL of washing buffer and then 100 µL/well of Human conjugate anti-Interleukin 8 antibody was added. The plate was incubated for one additional hour at room temperature, and after washing with 200 µL/well of washing Buffer, the reaction was revealed by adding 200 µL of substrate solution to each well and reading in a 450 nm plate reader.

### Parasites

*Leishmania amazonensis* (WHOM/BR/75/Josefa) promastigotes were maintained at 26 °C in Schneider’s Insect medium (Sigma) supplemented with 10% heat-inactivated fetal calf serum, 10% human urine, and 40 µg/mL of gentamicin. Stationary-phase promastigotes were obtained from 5- to 6-day-old cultures, and used throughout.

### Immunoflorescence

Purified neutrophils (2 × 10^5^ cells/mL) were plated on 0.001% L-polylysine-treated coverslips (24-well plate) in RPMI medium containing 1% nutridoma (Sigma-Aldrich). The cells were subjected with different stimuli (100 nM PMA, active or heat-inactivated virus) for 90 min at 37 °C/5% CO_2_. Afterwards, the coverslips were washed with PBS and fixed in 4% paraformoldehyde for 20 min, followed by blocking for 1 h (PBS containing 0.05% Tween 0.2 M glycine, 10% BSA, 0.01% gelatin and 0.1% triton). The staining was carried out using primary antibody (1:50) for anti-rabbit myeloperoxidase (Abcam 9535) diluted in PBS 0.05% Tween containing 3% BSA, 0.01% gelatin, 0.1% triton and 1% normal sheep serum for overnight at 4 °C. After washing with PBS 0.25% Tween, the reaction was developed using an appropriate secondary antibody (1: 150, Sigma-Aldrich) for 1 h, following incubation with Hoechst 33342 (2ʹ- [4-ethoxyphenyl] -5- [4 -methyl-1-piperazinyl] -2,5′-bi-1H-benzimidazole trihydrochloride trihydrate), a cell-permeable DNA stain (Thermofisher), for 10 min at room temperature. The coverslips were placed on slides containing approximately 10 µL of Vectachield (Sigma-Aldrich) for analysis using an Olympus BX51 fluorescence microscope.

### Data analysis

Results are expressed as mean ± SEM and p ≤ 0.05 was considered significant. For multiple comparisons, One-way ANOVA analysis followed by Tukey’s least significant difference was performed. Paired t-test analysis was performed for some experiments as indicated in the figure legend. Data analysis was performed by using the GraphPad Prism 5.03 software.

## Results

**Figure 1:**
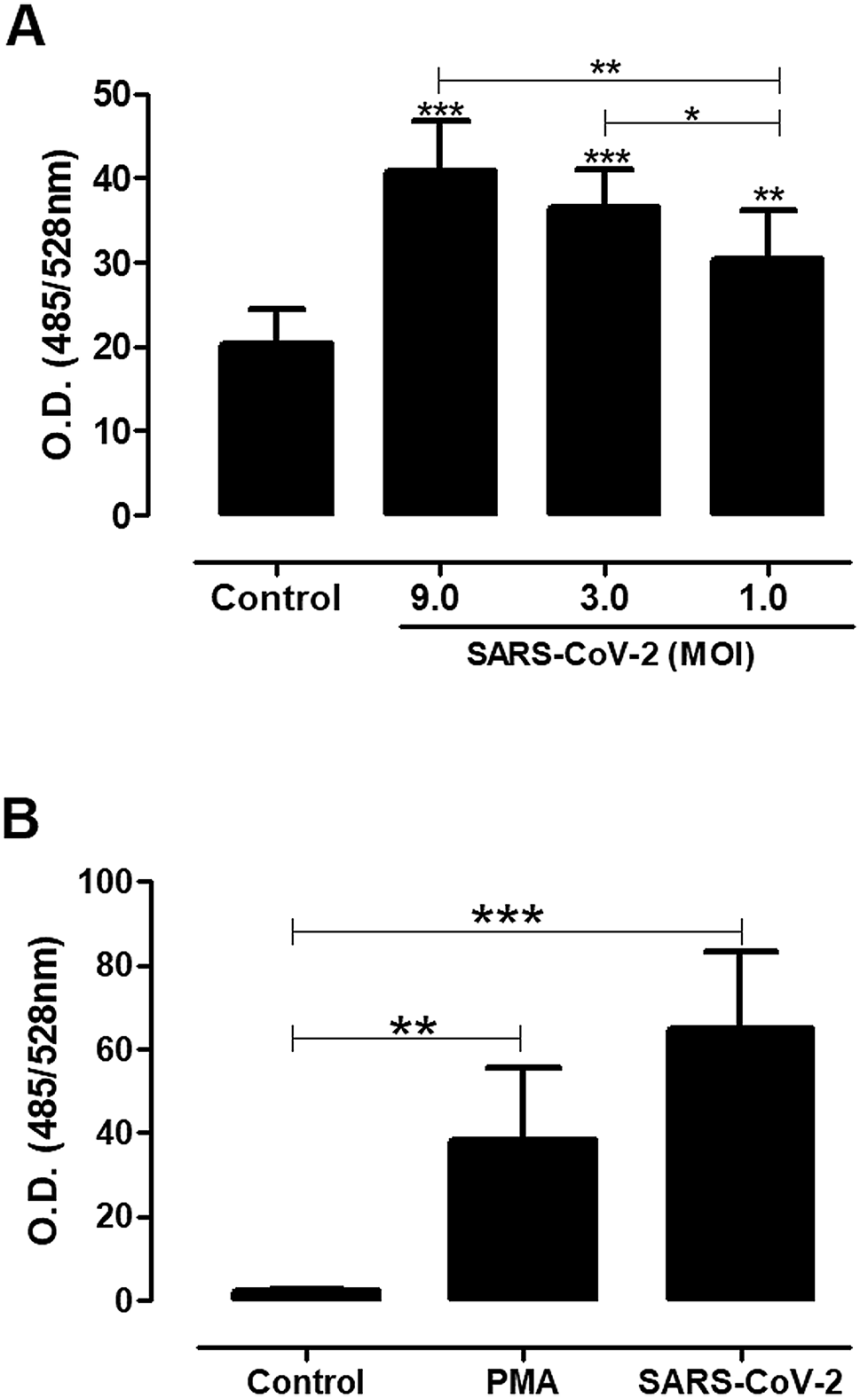
Neutrophils release extracellular DNA in response to coronavirus 2 (SARS-CoV-2). Neutrophils (5 × 10^4^/well) were stimulated or not with SARS-CoV-2 at different MOI ratios (**A**) or fixed MOI of 9.0 (**B**). The cells were incubated with virus or PMA (positive control) and after 90 min the supernatants were collected and the extracellular DNA release quantified by the Quant-iT PicoGreen dsDNA method, specific reagent for double-stranded DNA labeling in optical density assay (528 nm). The baseline control autofluorescence for the excitation/emission wavelengths (485/538 nm) is 6.30 ± 2.14. The data are representative of three independent experiments. The results were analyzed using One-way ANOVA followed by Tukey’s. Differences between groups are significant *p ≤ 0.05, **p ≤ 0.0025, ***p ≤ 0.0001.

We sought to investigate whether SARS-CoV-2 virus could activate neutrophils to induce NETosis. Although there is no evidence that viruses can use neutrophils to establish productive infections, many viruses can be detected within neutrophils, being even able to activate neutrophils to produce NETs. However, the exact mechanisms underlying this phenomenon are not fully characterized, it is possible that this process occurs via pattern recognition receptors (PRRs) present in endosomes or even expressed on the surface of neutrophils^16^. To assess the induction of NETosis by SARS-CoV-2, human neutrophils were stimulated with virus at MOI of 9.0 and the release of DNA was measured 90 min later. We strikingly found that SARS-CoV-2 was able to induce a significant increase of DNA released to supernatant in the presence of virus as compared to spontaneous release from negative control. We did not find any statistical differences between the MOI of 9.0 and 3.0, both being significantly higher than MOI of 1.0 (Fig. 1A). Additionally, in a control experiment for rapid NETs release, neutrophils were incubated with PMA for the same time point. Our results show that SARS-CoV-2 induced the classical NETosis at comparable levels of PMA, after 90 min of incubation (Fig. 1B).

**Figure 2:**
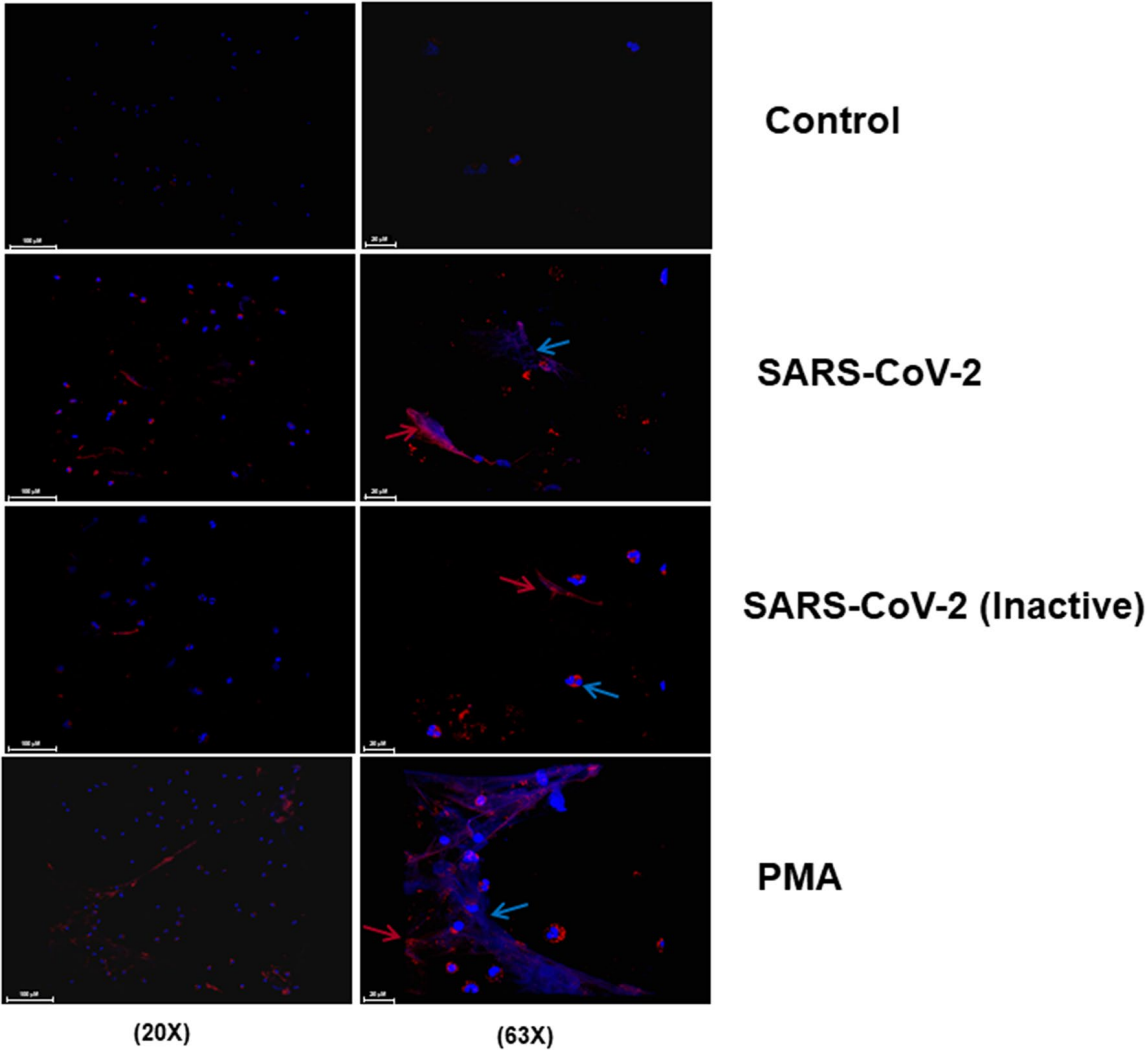
Active coronavirus 2 (SARS-CoV-2) potentiates the activation of neutrophil extracellular traps (NETs) formation. Neutrophils (5 × 10^4^/well) were stimulated alternatively with live or heat-inactivated SARS-CoV-2 (MOI of 9.0), and PMA (positive control). After 90 min of incubation, the coverslips were probed using anti-rabbit myeloperoxidase (Abcam 9535) and HOECHST (DNA marker). Arrows indicate the formation of structural NETs, showing the DNA (blue) and myeloperoxidase (red) markers. The images were made using an Olympus BX51 fluorescence microscope.

**Figure 3:**
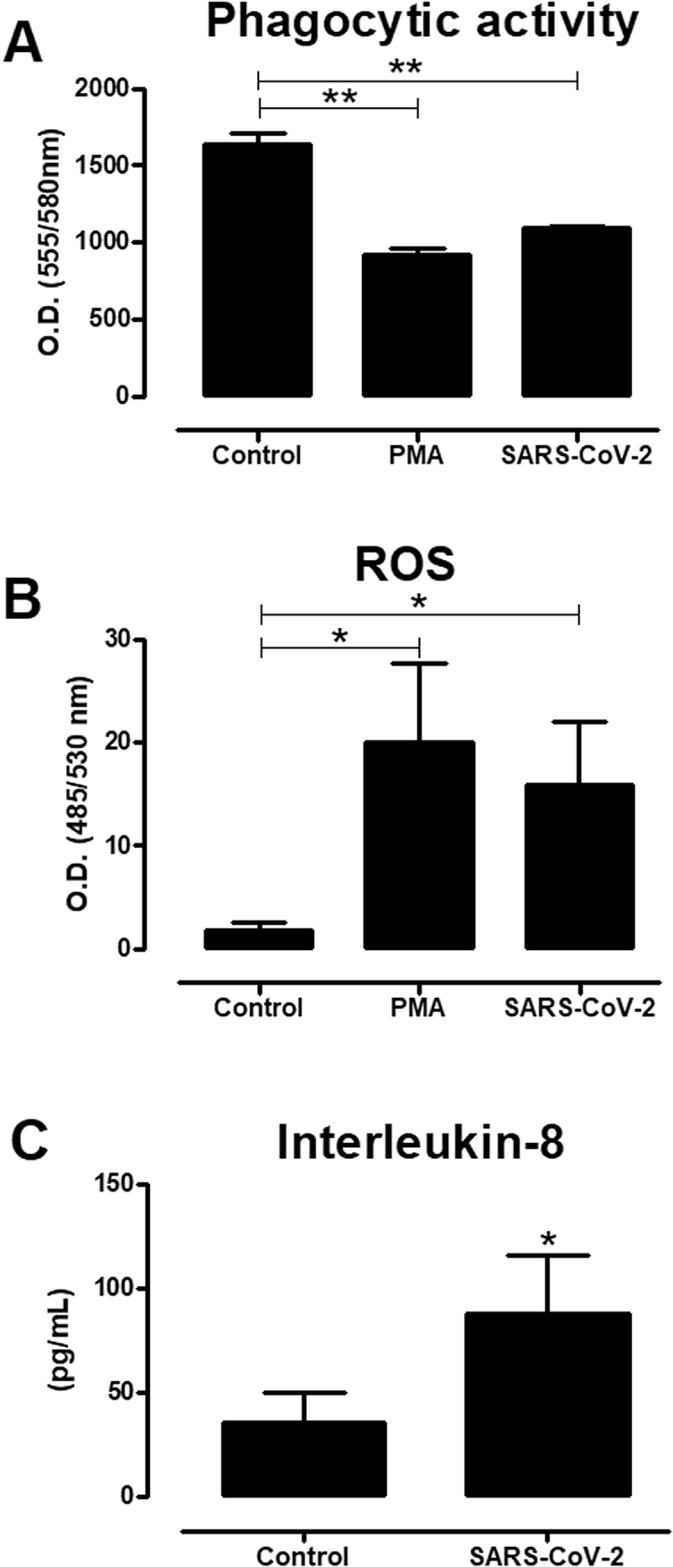
Neutrophils activate reactive oxygen species (ROS) and IL-8 secretion in response to coronavirus 2 (SARS-CoV-2). Neutrophils (5 × 10^4^/well) were stimulated or not with SARS-CoV-2 virus (MOI of 9.0) or PMA (positive control), and after 90 min of incubation the quantification of reactive oxygen species (ROS) was determined (**A**). Intracellular ROS was quantified using H2DCFDA probe (see “Materials and methods”), and the values expressed refer to the quantification of florescence incorporated by the probe (530 nm). (**B**) The phagocytic activity was analyzed by uptake of Dextran beads conjugated to tetramethyl rhodamine (2,000,000 MW) added to the cells (200 µg/mL) in the presence of the stimuli. The values, expressed in optical density (580 nm), refer to the incorporated florescence after 90 min of incubation. (**C**) The quantification of interleukin-8 (IL-8) production from neutrophils stimulated with SARS-CoV-2 virus (MOI of 9.0) was measured from culture supernatants collected after a 90-min incubation according to the recommendations of the manufacturer Quantikine Elisa/R&D System (see “Materials and methods”). The baseline control autofluorescence for the excitation/emission wavelengths 485/530 nm is 3.43 ± 1.79; and 555/580 nm is 348.85 ± 112.03. The results were analyzed using paired t-test analysis and One-way ANOVA followed by Tukey’s. Differences between groups are significant *p ≤ 0.05, **p ≤ 0.0025.

Further studies indicate that these findings are corroborated by the evidence of myeloperoxidase staining in neutrophils treated with virus, a classic marker of NETs^17^. This staining was reduced when the virus was heat-inactivated (60 °C/30 min), indicating the importance of viral activity for NETosis (Fig. 2). Besides their ability to control infection of pathogens by inducing NETosis, neutrophils have a plethora of defense mechanisms. In the human blood, neutrophils are the prevalent phagocytic cells, accounting for about 50% of all leukocytes. They are the major phagocyte of the innate immunity and plays a key role in the host defense against infectious pathogens^18,19^. We next addressed whether the SARS-CoV-2 could activate the phagocytic capacity of neutrophils. To approach this issue, we used dextran beads conjugated to fluorescein as a marker of phagocytosis. Human neutrophils were plated in monolayers in 96-well plates and stimulated with SARS-CoV-2 virus at MOI of 9.0 in the presence of fluorescein-labeled dextran beads. Phagocytosis was assessed 90 min later by measuring the intracellular neutrophil uptake. Our results indicate a reduction in the incorporation of beads in cells treated with SARS-CoV-2, as compared to the phagocytic activity of control neutrophils. This magnitude of reduction was observed in neutrophils treated with PMA for the same time point, indicating that NETosis in these groups partially impaired the ability of the cells to phagocyte (Fig. 3A).

Next we tested whether SARS-CoV-2 could induce neutrophilic Reactive Oxygen Species (ROS). ROS generated by NADPH oxidase play an important role in the clearance of RNA virus by neutrophils. These cells release large amounts of ROS at the site of infection following the activation of G-protein-coupled receptors (GPCRs), toll-like receptors or IL-8-induced priming of the oxidative burst. ROS released by the NADPH oxidase complex can also activate granular proteases to induce NETosis^19,20^. Our findings demonstrate that neutrophils are capable of inducing the production and release of ROS when incubated with SARS-CoV-2 for 90 min (MOI of 9.0). The same response was observed in the positive controls treated with PMA (Fig. 3B). Furthermore, neutrophils have a preformed capacity to produce the cytokine IL-8, considering that IL-8 mRNA is produced constitutively by these cells. The production of this proinflammatory cytokine could have a possible autocrine effect in stimulating ROS by neutrophils^20^, as our results indicate that treatment with SARS-CoV-2 induced a modulation in the levels of secreted IL-8, when compared to other control groups (Fig. 3C).

**Figure 4:**
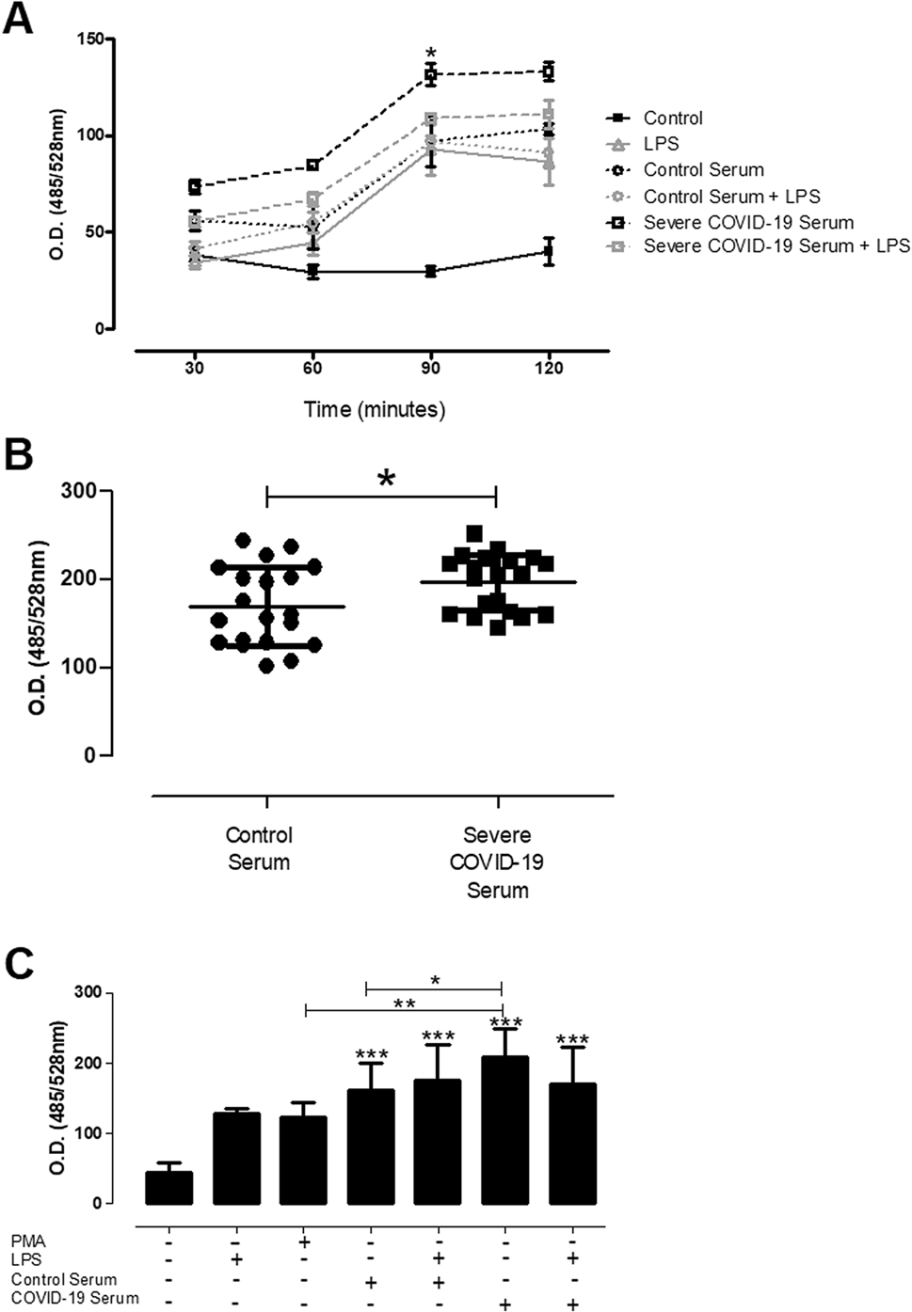
Neutrophils release extracellular DNA in response to sera from patients with severe acute respiratory syndrome coronavirus 2 (SARS-CoV-2) infection. Neutrophils (5 × 10^4^/well) were stimulated or not with LPS (10 µg/mL) in the presence of 10% sera from normal donors or severe patients in the acute phase of infection, after the different stimulation times indicated in the kinetics (**A**), or at a time point of 90 min (**B**,**C**)**.** The supernatants were then collected and NETs were quantified by the Quant-iT PicoGreen dsDNA method, using specific reagent for double-stranded DNA detection by optical density (528 nm). The data are representative of three independent experiments. (**A**) Sera from two donors (healthy control and severe COVID-19 patient) were used; (**B**,**C**) Sera from a cohort of 40 donors (20 healthy controls and severe 20 severe COVID-19 patients in the acute phase of infection) were used. The results were analyzed using paired t-test analysis and One-way ANOVA followed by Tukey’s. Differences between groups are significant *p ≤ 0.05, **p ≤ 0.0025.

Detection of active viral particles in the peripheral blood is linked to disease severity^21–23^. Sera derived from these patients presents several thrombolytic factors and inflammatory cytokines that could activate NETosis in neutrophils. According to our hypothesis, our results demonstrated that addition of sera from severe COVID-19 patients (Table 1) to neutrophil monolayer induced the release of DNA compatible with NETs, as compared with heterologous control sera. Our results indicate that this increase was progressive over the 120 min of kinetics analyzed. Both serum groups had a higher DNA release index than spontaneous release of negative controls. The addition of LPS (Supplementary Fig. 1) to sera did not confer any synergistic effect on DNA release by activated neutrophils (Fig. 4A). In a larger cohort, using 20 individuals per group, our data corroborate the previous analysis showing a significant increase in the release of DNA by neutrophils treated with sera obtained from severe COVID-19 patients when compared with normal sera, used as controls (Fig. 4B).

**Figure 5:**
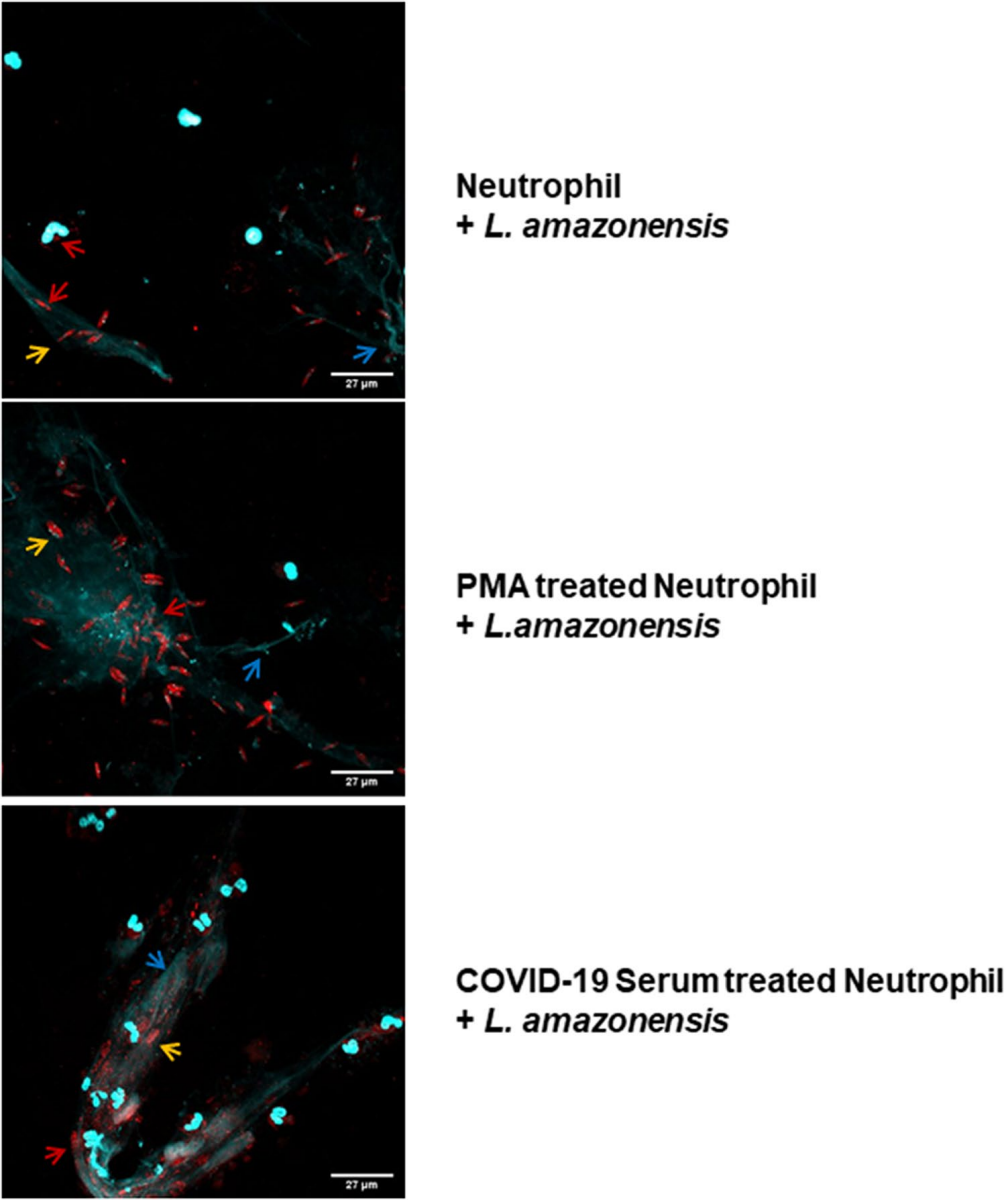
Neutrophil extracellular trap activated in COVID-19 are structurally and functionally active with ability to mediate pathogen entrapment. Neutrophils (2 × 10^5^) were incubated with 100 nM PMA (Sigma) or 10% serum from severe COVID-19 patients in the acute phase of infection for 90 min. Afterwards, *Leishmania amazonensis* promastigotes were added to the culture (10 parasites per neutrophil) and incubated for 2 h at 35 °C for immunocytochemistry analysis. The coverslips were probed using anti-rabbit myeloperoxidase (Abcam 9535) and HOECHST (DNA marker). Arrows indicate the formation of structural NETs, showing the DNA (blue) and myeloperoxidase (red) markers. Yellow arrows indicate the presence of entrapped Leishmania parasites. The images were made using an Olympus BX51 fluorescence microscope.

In an independent experimental set using this cohort, we showed that the neutrophil response to COVID-19 serum was not synergistically modulated by the addition of LPS (Fig. 4C). Immunocytochemistry analysis by staining myeloperoxidase and extracellular DNA indicates the deposition of structural NETs (Fig. 5). We further investigated whether this NET is actually functional in terms of its microorganism trapping capability. For this purpose, we use as a sentinel pathogen of its action, the *Leishmania* parasite, a pathogen that presents stages with flagellar motility and is known to activate NETosis^24^. In our experimental approach, neutrophils were activated with COVID-19 serum or PMA as positive control, and after 90 min the cultures were treated with motile promastigote forms of *Leishmania* parasites. After fixation and labeling with fluorescent nucleic acid dye picogreen for visualization of the double-stranded DNA neutrophilic network, our results demonstrated the functional capacity of NETs induced by the serum from COVID-19 critically infected patients in trapping live parasites, compared to PMA positive controls (Fig. 5). The parasites trapped in the neutrophilic network activated by the COVID-19 serum showed loss of integrity probably due to the action of the complement system.

## Discussion

The ongoing pandemic COVID-19 is a respiratory viral infection associated in about 20% of individuals infected with severe acute respiratory syndrome, predisposing to thrombosis both in veins and arteries due to excessive inflammation, platelet activation, endothelial dysfunction and stasis. The most frequent hemostatic change due to infection is thrombocytopenia and elevation of D-dimer^7^. In Case–control studies using a cohort of 183 patients it was found that the degree of activation of D-dimers was much higher in patients who did not survive than in those who survived. In addition, in 71% of those who died, clots were found. The increase of D-dimer was correlated to an increased need for invasive ventilation, intensive care and death^7,21^. It is still unclear whether these changes are direct consequences of viral virulence or the effect of the systemic inflammatory response. Recent studies have shown that many critically infected patients admitted with COVID-19 exhibit generalized microthrombi formed in veins or arteries, and that can be directly related to the severity and lethality of the disease. These studies revealed that 31% of 184 patients hospitalized for the new pneumonia virus had abnormally clotted blood, a percentage considered extraordinarily high, as compared to hospitalization for others causes^25^.

Despite all the data converging to a strong relationship between the new coronavirus and the formation of microthrombus, the mechanisms leading this process remains incompletely understood. It has been revealed that severe COVID-19 patients share common characteristics to the development of Acute Respiratory Distress Syndrome with the presence of thick mucus secretions in the airways and the development of blood clots^26^. These symptoms are similar to those of diseases already known as being caused by NETs, including chronic obstructive pulmonary disease and pneumonia induced by pathogen infections. The pathological effect of NETs is not restricted to airway obstruction in lung injuries, but also play a role in the occlusion of arteries and vessels in degenerative cardiovascular disease^14^. Recent studies have demonstrated elevated levels of NET markers such as cell-free DNA, myeloperoxidase (MPO)-DNA, and citrullinated histone H3 (Cit-H3) in serum samples from patients with severe COVID-19, but not in healthy controls^27^. In such a study, the authors showed that the sera from infected patients could trigger NET formation by healthy neutrophils. This notion was corroborated in our study using a cohort of 20 severe COVID-19 patients with acute respiratory distress syndrome. We showed that the neutrophilic network activated by the serum from patients in the severe form of the disease is structurally and functionally active, in view of its ability to induce extracellular NET-mediated entrapment of pathogens, such as *Leishmania* parasite. It is possible that the activation of NET in these sera is due to the presence of active virus in the blood of patients in the severe form of the disease^22,23^.

We further showed for the first time that SARS-CoV-2 virus is able to activate NETosis in human neutrophils. Furthermore, we demonstrated that this process is associated with increased levels of intracellular Reactive Oxygen Species (ROS) of neutrophils incubated in the presence of SARS-CoV-2. Reactive oxygen species can kill pathogens directly by causing oxidative damage or indirectly, in neutrophils, by stimulating pathogen elimination via extracellular neutrophil trap formation^16,19^. ROS also has a detrimental role, promoting venous thrombus formation through the modulation of the enzymatic cascade of fibrinolysis systems of coagulation and the complement system^28^. These findings undoubtedly point to a critical role for neutrophils in the pathology of infection. It will be important to determine whether the presence of NETs in immunohistochemistry analysis of lung tissue from autopsy samples are associated with disease severity and/or particular clinical characteristics of COVID-19. In other severe or persistent viral infections, neutrophil-mediated alveolar damage leads to interstitial edema, ventilation/perfusion mismatch and respiratory failure. Recent studies have identified neutrophil infiltration in the pulmonary capillaries in autopsy reports of COVID-19 patients^29^. This further supports the hypothesis that neutrophils may be responsible for the severity of the disease.

Taking into account that the severity of the SARS-CoV-2 infection has linked to NET formation, it is possible that compounds that degrade NETs or block their formation could relieve ARDS associated with disease. This is seen in cases of cystic fibrosis in which the therapeutic use of dornase alfa, which dissolves NETs by cleaving DNA, and provides loosen sputum and relieve symptoms^30^. The protocol for administering these medications cannot be organ-specific since the disease has been revealed as a systemic disease. The upregulation of ACE2 in patients with comorbidities may represent a propensity for an increased viral load and spreading of infection to extrapulmonary tissues^4,5^. The systemic infection would lead to a storm of pro-inflammatory cytokines leading to an extensive microthrombus formation with multiorgan failure in severe patients. As an alternative to this therapeutic target, studies have revealed that the use of heparin, a low-cost anticoagulant, was associated with an improvement in the prognosis of severe cases in COVID-19 resulting in increased oxygen levels in the patients’ blood^31^. Our studies also suggest the importance of using antioxidant treatments aiming at abrogating the possible participation of ROS generated by thrombosis in neutrophils activated by the SARS-CoV-2 infection. Further clinical studies are, however, needed to bring clarity to this issue. Despite the limitations in our capacity in responding promptly to the clinical demand for this disease, a comprehensive understanding of the viral pathogenesis is needed in order to eliminate this devastating pandemic virus.

### Substantial significance statement

Coronaviruses are a large family of viruses that cause diseases ranging from the common cold to more serious infections, such as the Middle East Respiratory Syndrome (MERS-CoV), the Severe Acute Respiratory Syndrome (SARS-CoV), and recently the new Coronavirus disease 2019 (COVID-19). Therefore, in the context of the global pandemic caused by SARS-CoV-2, there is an urgent need to better understand the pathophysiology of COVID-19. Herein, we demonstrated for the first time that SARS-CoV-2 stimulates extracellular neutrophils traps (NETs) in a process called NETosis. Our findings indicate that this process is associated with increased levels of intracellular Reactive Oxygen Species (ROS) in neutrophils. The ROS-NET pathway plays a role in thrombosis formation and our study suggest the importance of this target for therapy approaches against disease.

## Supplementary information

Supplementary Information
