# Supplementary material for: The emerging role of neutrophil extracellular traps in severe acute respiratory syndrome coronavirus 2 (COVID-19)
Source: Sci Rep. 2020 Nov 12;10:19630. doi: 10.1038/s41598-020-76781-0 (PMC7665044; doi:10.1038/s41598-020-76781-0)
Supplement: Supplementary file 1 — Supplementary Information [file 41598_2020_76781_MOESM1_ESM.docx]

**TITLE: The Emerging Role of Neutrophil Extracellular Traps in Severe Acute Respiratory Syndrome Coronavirus 2 (COVID-19)**

**Authors: Angélica Arcanjo, Jorgete Logullo, Camilla Cristie Barreto Menezes, Thais Chrispim de Souza Carvalho Giangiarulo, Mirella Carneiro dos Reis, Gabriellen Menezes Migliani de Castro, Yasmin da Silva Fontes, Adriane Regina Todeschini, Leonardo Freire-de-Lima, Debora Decoté-Ricardo, Antônio Ferreira-Pereira, Celio Geraldo Freire-de-Lima, Shana Priscila Coutinho Barroso, Christina Takiya, Fátima Conceição-Silva, Wilson Savino, Alexandre Morrot**


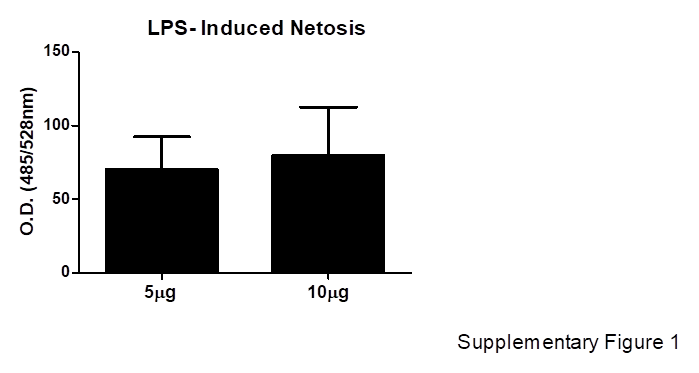


**Supplementary Figure 1. LPS-induced neutrophil extracellular traps (NETs) formation.** Neutrophils (5 x 10^4^/well) were stimulated with two different concentrations of LPS (5 or 10 µg/mL) to standardize the NETosis assay as referenced in other studies (32). After 90 minutes, the supernatants were collected and the NETs were quantified by the Quant-iT PicoGreen dsDNA method, using specific reagent for DNA detection double tape by optical density (528 nm). The data represent the optical densities obtained in each LPS value subtracted from the negative control with medium alone. The results are representative of three independent experiments.
